# Rs12218 In SAA1 gene was associated with serum lipid levels

**DOI:** 10.1186/1476-511X-12-116

**Published:** 2013-07-30

**Authors:** Xiao-Lin Xu, Xiao-Tian Sun, Liewen Pang, Guoqian Huang, Jiechun Huang, Meng Shi, Yi-Qing Wang

**Affiliations:** 1 Department of Cardiothoracic Surgery, Huashan Hospital, Fudan University, Shanghai 200032, PR China

**Keywords:** SAA1, Genetic polymorphism, Triglyceride, Total cholesterol, Low-density lipoprotein

## Abstract

**Background:**

Serum amyloid A (SAA) is a kind of apolipoprotein. Several studies indicated that SAA genetic polymorphism rs12218 was associated with carotid atherosclerosis, peripheral arterial disease, and serum uric acid levels. However, the relation between rs12218 and lipid levels remains unclear. This study assessed the correlation between SAA1 gene rs12218 polymorphism and lipid levels in a Chinese population.

**Methods:**

A total of 823 participants were selected from the subjects for health check in Shanghai Huashan hospital from Jan. 2013 to Mach. 2013. Correlations between rs12218 polymorphism and lipid levels were investigated through the identification of rs12218 genotypes using the polymerase chain reaction-restriction fragment length polymorphism (PCR-RFLP).

**Results:**

We found that the SNP rs12218 was associated with triglyceride (TG), total cholesterol (TC), and low-density lipoprotein (LDL-C) levels by analyses of a dominant model (*P*<0.001, P=0.002, P=0.003, respectively), a recessive model (*P* <0.001, *P*=0.001, *P*=0.005, respectively) and an additive model (*P* < 0.001, *P*=0.001, *P*=0.002, respectively), and the difference remained significant after the adjustment of sex, age, alcohol intake, and smoking (All *P* < 0.01).

**Conclusion:**

Our results indicated that the rs12218 in the *SAA1*gene was associated with lipid levels in a Chinese population.

## Background

Serum amyloid A (SAA) is a kind of apolipoprotein and is primarily synthesized in the liver by activated monocytes and macrophages [1]. As an apolipoprotein, SAA is associated with lipid level, such as high-density lipoprotein (HDL-C), and during inflammation can contribute up to 80% of its apoprotein composition [2].

The human SAA gene cluster on the short arm of chromosome 11, localized to band p15.1, contains four related genes, SAA1-4, within a 150-kb region [3]. Only the SAA1 and SAA2 genes encode acute-phase SAAs (SAA1 and SAA2 proteins), so a lot of research focuses on them. Recently, Carty et al. [4] reported an association of SAA1 and SAA2 gene polymorphisms and carotid intima-media thickness (cIMT), HDL-C, and total CVD. Many studies have demonstrated that rs12218 in the SAA1 gene was associated with carotid atherosclerosis [5] and peripheral arterial disease [6]. However, the relationships between SAA gene polymorphism and lipid level remain unclear.

In the present study, we aim to study the relationship between SAA1 gene polymorphism (rs12218) and lipids levels.

## Results and discussion

This study consists of 823 subjects. The clinical and metabolic characteristics of the study population are shown in Table 1.

**Table 1:** Characteristics of subjects

**Risk factors**	**No. (%) or Mean±SD**	
	**Male (n=654)**	**Female (n=169)**
Age (years)	53.2±11.8	54.1±10.7
Never drink (%)	421 (64.4)	118 (69.8)
Former drinker (%)	201(30.7)	32 (18.9)
Current drinker (%)	32 (4.9)	19 (11.2)
Never smoking (%)	452 (69.1)	131 (77.5)
Former smoking (%)	143 (21.9)	26 (15.4)
Current smoking (%)	59 (9.0)	12 (7.1)
BMI (Kg/m^2^)	24.4 ± 3.6	24.7 ± 3.9
SBP (mmHg)	124.4 ± 13.3	120.6 ± 10.1
DBP (mmHg)	78.7 ± 10.6	76.6 ± 7.4
GLU (mmol/L)	4.55 ± 0.81	4.43 ± 0.41
TG (mmol/L)	0.97 ± 0.37	0.99 ± 0.38
TC (mmol/L)	4.33 ± 0.91	4.18 ± 0.91
HDL (mmol/L)	1.03 ± 0.44	1.17 ±0.43
LDL-C (mmol/L)	2.68 ± 0.84	2.59±0.81

The distribution of genotype was in Hardy-Weinberg equilibrium (*P*>0.05, data not shown). Table 2 shows detailed information for rs12218 as well as the allele frequencies.

**Table 2:** Distribution of genotypes and alleles

**Genotypes, n, %**	**CC**	**CT**	**TT**
	129 (15.7)	332 (40.3)	362 (44.0)
Alleles, n, %	C		T
	590 (35.8)		1056 (64.2)

We found that the SNP rs12218 was associated with triglyceride (TG), total cholesterol (TC), and low-density lipoprotein (LDL-C) levels by analyses of a dominant model (*P*<0.001, P=0.002, P=0.003, respectively), recessive model (*P* <0.001, *P*=0.001, *P*=0.005, respectively) and additive model (*P* < 0.001, *P*=0.001, *P*=0.002, respectively), and the difference remained significant after the adjustment of sex, age, alcohol intake, and smoking (All *P* < 0.01) (Table 3).

**Table 3:** Association of rs12218 with lipid levels

	**Rs12218**			**Model 1‡**			**Model 2§**		
Lipid profile	CC	CT	TT	*P*	*P*	*P*	*P*	*P*	*P*
				Rec*	Dom†	Add^※^	Rec*	Dom†	Add^※^
TG (mmol/L)	0.72 ± 0.32	0.96±0.34	1.01 ±0.32	**<0.001**	**<0.001**	**<0.001**	**<0.001**	**<0.001**	**<0.001**
TC (mmol/L)	3.94 ± 1.10	4.18 ± 0.94	4.32 ± 0.94	0.003	0.002	0.001	0.001	0.002	0.001
HDL-C (mmol/L)	1.26 ± 0.42	1.29 ± 0.45	1.31 ± 0.44	0.346	0.473	0.596	0.324	0.132	0.435
LDL-C (mmol/L)	2.43 ± 0.86	2.58± 0.79	2.70 ± 0.80	0.005	0.003	0.002	0.012	<0.001	0.002

§analysis of covariance adjusted for sex, age, smoking, alcohol drinking, and GLU; ‡Unadjusted model; *recessive model; †dominant model; ^※^additive model.

In the present study, we found that variation in the SAA1 gene is associated with TG, TC, and LDL-C levels in Chinese population. In the early 1970s, SAA was identified as the plasma protein responsible for forming tissue deposits called “amyloid (AA-type)” seen in diseases with underlying persistent acute inflammation [7,8]. Soon after its discovery, SAA was shown to be an acute phase protein produced by the liver within hours of tissue injury regardless of cause. Its plasma concentration can increase a 1000-fold within 24 h [9,10]. In plasma, SAA is associated with HDL-C [11,12] and, during severe inflammation, can contribute 80% of its apo-protein composition [13]. The displaced apoA-I is rapidly cleared by the liver and kidneys [14], together with a sharp decline in apoA-I gene expression during inflammation [15]. A relationship between the SAA1 gene polymorphism and cardiovascular diseases has been reported previously [1,2,4]. Previous studies have investigated the SAA1 rs12218 polymorphism in the Chinese population, but its relationship with lipid level has not been thoroughly investigated. Xie et al. [5] and Feng et al. [1] reported the relationship between rs12218 and lipid level, however, they did not reach to the same conclusion. Feng et al. reported an association between HDL-C concentration and rs12218, he found that in the osteoporosis group the rs12218 was significantly associated with plasma TC, HDL-C, and LDL-C levels (P=0.021, P=0.009, and P=0.009, respectively). However, this association was not found in the control group. And, they did not find the TG level was significantly associated with rs12218. Xie et al. also found rs12218 was significantly associated with HDL-C concentration, but they did not found the TG, LDL-C and TC was associated with rs12218. In the present study, we found rs12218 associated with TG, TC and LDL-C level both in the additive model, but also in dominant model and recessive model. After adjusted for some confounders the difference remains significant. This discrepancy may be result from the different population selection. And the lager-sample study related to this issue was expected.

## Conclusions

In conclusion, the SAA1 gene polymorphism was associated with lipid level in a Chinese population.

## Subjects and methods

### Subjects

This study was approved by the Ethics Committee of the Shanghai Fudan University and was conducted according to the standards of the Declaration of Helsinki. Written informed consent was obtained from the participants. A total of 823 participants were selected from the subjects for health check in our hospital from Jan. 2013 to Mach. 2013 in Shanghai Huashan Hospital. These subjects were free from diabetes, hypertension, or any history of coronary artery disease (CAD). Height, body weight, and blood pressure were measured as described previously [16,17]. Smoking and drinking status was self-reported by study questionnaire as described previously [17]. We measured the fasting plasma concentration of total cholesterol, triglyceride (TG), low-density lipoprotein (LDL), high-density lipoprotein (HDL) and glucose using an equipment for chemical analysis (Dimension AR/AVL Clinical Chemistry System, Newark, NJ) employed by the Clinical Laboratory Department of Shanghai Huashan hospital.

### Rs12218 genotyping

Genomic DNA was extracted from the peripheral blood leukocytes using a DNA extraction Kit (Beijing Bioteke Co. Ltd, China). We genotyped rs12218 according to the protocol described previously [2]. To ensure the results were verified, 10% of the genotyped samples were duplicated, and at least one positive and one negative control per 96-well DNA plate were used in our assays. The accuracy of the genotyping was determined by assessing the genotype concordance between duplicate samples. We obtained a 100% concordance between the genotyped duplicate samples for the SNP. The genotyping success rate was 100%.

### Statistical analysis

All analyses were carried out using SPSS version 17.0 (SPSS Inc., Chicago, IL, USA). The Hardy-Weinberg equilibrium was assessed using chi-square analysis. The characteristics of the study population were expressed as the mean ± standard deviation or as a ratio. Fasting triglycerides were log-transformed using natural logarithms for analysis. General linear model analysis was undertaken to test for associations between SNP genotypes and lipid levels after adjusting for confounding variables. Single-SNP effects with continuous variables were analyzed using linear regression using three models. These were the additive (common allele homozygotes coded as 1, heterozygotes as 2, and recessive allele homozygotes as 3); dominant (common allele homozygotes coded as 1 and heterozygotes and recessive allele homozygotesas 2); and recessive (common allele homozygotes and heterozygotes coded as1 and recessive allele homozygotes as 2) models as described previously [16]. Normality was assessed by plotting the residuals.

## Abbreviations

SAA: Serum amyloid A; CAD: Coronary artery disease; TG: Triglycerides; TC: Total cholesterol; HDL-C: High-density lipoprotein; LDL-C: Low-density lipoprotein.

## Competing interests

The author declare that they have no competing interests.

## Authors’ contributions

XLX and XTS carried out the molecular genetic studies and drafted the manuscript. LWP and GQH carried out the genotyping. JCH, XTS and XLX participated in the design of the study and performed the statistical analysis. MS and YQW conceived of the study, and participated in its design and coordination and helped to draft the manuscript. All authors read and approved the final manuscript.
